# Feasibility of delta radiomics–based pCR prediction for rectal cancer patients treated with magnetic resonance–guided adaptive radiotherapy

**DOI:** 10.3389/fonc.2023.1230519

**Published:** 2023-11-24

**Authors:** Junxiang Wu, Juan Xiao, Yihong Li, Fan Wu, Qian Peng, Churong Li, Bin Tang, Lucia Clara Orlandini

**Affiliations:** ^1^ Radiation Oncology Department Sichuan Cancer Hospital and Institute, Affiliated Cancer Hospital of University of Electronic Science and Technology of China, Sichuan Clinical Research Center for Cancer, Chengdu, China; ^2^ School of Medicine, University of Electronic Science and Technology of China, Chengdu, China; ^3^ Clinical Medical College, Chengdu University of Traditional Chinese Medicine, Chengdu, China

**Keywords:** delta radiomics, pathological complete response, rectal cancer, MRgART, neoadjuvant chemoradiotherapy, MR-Linac

## Abstract

Magnetic resonance–guided adaptive radiotherapy (MRgART) represents the latest frontier in precision radiotherapy. It is distinguished from other modalities by the possibility of acquiring high-contrast soft tissue images, combined with the ability to recalculate and re-optimize the plan on the daily anatomy. The extensive database of available images offers ample scope for using disciplines such as radiomics to try to correlate features and outcomes. This study aimed to correlate the change of radiomics feature along the treatment to pathological complete response (pCR) for locally advanced rectal cancer (LARC) patients. Twenty-eight LARC patients undergoing neoadjuvant chemoradiotherapy (nCRT) with a short course (25 Gy, 5 Gy × 5f) MRgART at 1.5 Tesla MR-Linac were enrolled. The T2-weighted images acquired at each fraction, corresponding target delineation, pCR result of the surgical specimen, and clinical variables were collected. Seven families of features [First Order, Shape, Gray-level Co-occurrence Matrix (GLCM), Gray-level Dependence Matrix (GLDM), Gray-level Run Length Matrix (GLRLM), Gray-level Size Zone Matrix (GLSZM), and Neighborhood Gray Tone Difference Matrix (NGTDM)] were extracted, and delta features were calculated from the ratio of features of each successive fraction to those of the first fraction. Mann-Whitney U test and LASSO were utilized to reduce the dimension of features and select those features that are most significant to pCR. At last, the radiomics signatures were established by linear regression with the final set of features and their coefficients. A total of 581 radiomics features were extracted, and 2,324 delta features were calculated for each patient. Nineteen features and delta features, and one clinical variable (cN) were significant (*p*< 0.05) to pCR; seven predictive features were further selected and included in the linear regression to construct the radiomics signature significantly discriminating pCR and non-pCR groups (*p*< 0.05). Delta features based on MRI images acquired during a short course MRgART could potentially be used to predict treatment response in LARC patients undergoing nCRT.

## Introduction

Colon-rectum cancer ranks as the second leading cause of cancer-related mortality worldwide (1, 2), representing approximately 9.4% (935,173) of all new cancer cases. Currently, the standard treatment approach for locally advanced rectal cancer (LARC) involves neoadjuvant radiochemotherapy (nCRT), followed by total mesorectal excision (TME) (3–7). nCRT induces a pathological complete response (pCR) in approximately 11%–42% of LARC patients (8). Extensive research has demonstrated that patients achieving pCR generally experience a more favorable prognosis, characterized by a reduced risk of local failure, lower propensity for local or distant recurrence, improved overall survival, and metastasis-free survival (9, 10). Recent studies have proposed conservative treatment strategies for patients who achieve clinical complete response (cCR) after nCRT, such as local excision or Watch and Wait (W&W), aiming to minimize morbidity and long-term effects associated with unnecessary TME surgery on anorectal and sexual function (11, 12). However, the concept of cCR remains controversial, as it relies on the absence of detectable tumor during clinical and radiological re-evaluation following nCRT. Notably, some research found all patients with pCR also exhibit cCR, not all patients with cCR achieve pCR (13); nevertheless, other studies found different results showing that 61.3% of patients with a pCR had evidence of non-cCR (14). Therefore, early identification of patients who will achieve pCR before TME surgery has become increasingly vital for their accurate multidisciplinary management and remains a topic of ongoing research. Radiomics is an emerging field that includes the extraction of concealed data from medical images and the development of the classification model by machine learning or deep learning techniques. This field aims to provide diagnostic and prognostic insights by detecting features of the tumor or healthy tissue that may not be easily discernible through visual examination (15, 16). Radiomics encompasses the extraction and analysis of spatial layout and geometric shape across various imaging techniques, including computed tomography (CT), magnetic resonance (MR), and positron emission tomography (PET) (17–20). Leveraging these characteristics, numerous studies (21–29) have employed radiomics based on clinical medical images to predict pCR or cCR in LARC, facilitating accurate diagnosis and appropriate treatment selection for patients. Magnetic resonance imaging (MRI) serves as the gold standard in rectal cancer diagnosis, delivering excellent soft tissue contrast and high spatial resolution for assessing LARC response (30). Consequently, several radiomics approaches have been applied to MRI, enabling the prediction of pCR or cCR response to nCRT (22, 31), long-term survival in LARC patients (32), and recognition of different stages of rectal cancer (33). In addition to classical radiomics, delta radiomics is also gaining ground (34); compared to radiomics, which is based on clinical images acquired at a single time point, delta radiomics studies the temporal variation of radiomic features extracted from a dataset of images acquired throughout the treatment. Multiple studies have proposed delta radiomics as an alternative approach for predicting treatment response, tumor prognosis, toxicity, and treatment guidance across various cancer types (35–41). In the specific field of LARC patients, delta radiomics has been proven effective in predicting the response of metastatic renal cell cancer and colorectal cancer liver metastases to chemotherapies (36, 42), cCR or pCR after nCRT (8, 43), disease-free overall survival (44). To the best of our knowledge, the application of delta radiomics based on MR-Linac imaging to predict pCR in LARC patients undergoing a short course of radiotherapy (SCRT) has not been explored previously.

Our study is focused on the treatment of LARC patients treated at 1.5 Tesla Magnetic Resonance Linac (MR-Linac). The use of such a hybrid machine enables a precise magnetic resonance-guided adaptive radiotherapy (MRgART) (45) by the acquisition of diagnostic-quality MRI scans before each daily fraction, facilitating accurate dose delivery, hypo-fractionated dose escalation to the tumor, and improving local control rates with reduced toxicity rates. Importantly, via the use of an MR-Linac, we can collect multiple MRI images throughout the treatment process and, therefore, there is ample room for delta radiomics analysis.

This study aims to predict the response of LARC patients undergoing a SCRT with a 1.5 Tesla MR-Linac. Specifically, we aim to assess the feasibility of using delta radiomics as a predictive tool to determine pCR of LARC patients after nCRT. By investigating the potential of delta radiomics, this research strives to enhance patient management and treatment outcomes.

## Materials and methods

### Patients

This retrospective study includes a cohort of 28 patients diagnosed with pathologically confirmed LARC. Specifically, patients with clinical stage T3-4N0 or T3-4N1-2 who received MRgART using a 1.5 Tesla MR-Linac were included in this retrospective study. To maintain the study’s focus, exclusion criteria comprised patients with distant metastases, a history of prior chemotherapy or radiotherapy for rectal cancer, concurrent or previous malignancies, and individuals with known allergies to intravenous contrast agents or contraindications to MRI. This study was approved by the Institutional Ethics Committee of our hospital.

### Patient clinical workflow

Patients underwent a SCRT followed after one week by chemotherapy, then evaluated as cCR or non-cCR according to the criteria described in the next section. In the absence of cCR, surgery was scheduled, otherwise, patients followed the W&W strategy with close follow-up consisting of regular restaging by MRI and digital rectal examination.

SCRT was administered over 1 week delivering 25 Gy in 5 Gy per fraction. Each treatment fraction starts with the acquisition of an online MRI used for the plan adaptation. The pre-treatment CT reference image, contours, and plan, together with the daily online MRI are used as input to adapt the plan for that specific session. The reference image is matched with the online MRI through rigid registration, then the workflow proceeds with an adapt to shape (ATS) approach based on the new patient anatomy; target and organs at risk were adapted to the daily anatomy using deformable registration with normalized mutual information of Monaco (Elekta AB, Stockholm, Sweden) v.5.4, and then manually adjusted (if needed) by the radiation oncologist. The CT reference treatment plan includes all the information needed to generate the synthetic CT (sCT) including average electron density and a given layering priority for each contour; the adapted plan is then recalculated on the daily MR-based sCT.

Chemotherapy was administered for 12 weeks, comprising six cycles of mFOLFOX6 regimen. This regimen involved intravenous administration of Oxaliplatin (85 mg/m^2^) and folinic acid (400 mg/m^2^) on day 1, intravenous push of 5-fluorouracil (400 mg/m^2^) on day 1, and continuous intravenous pumping of 5-fluorouracil (2400 mg/m^2^) over 46h–48h, repeated every 14 days.

TME surgery was performed through either anterior resection or abdominoperineal resection, depending on the individual patient’s requirements and circumstances.

### cCR and pCR evaluation criteria

In this study, a patient was considered as cCR when the following criteria were fulfilled: absence of lymph nodes on restaging MRI imaging, no residual primary tumor evident on morphological and diffusion-weighted imaging with intact rectal wall layers, absence of detectable mass during manual rectal examination, and no residual lesions or presence of a flat scar during endoscopic examination.

The pathologic staging was determined following the tumor node metastasis (TNM) classification system, recommended by the American Joint Committee on Cancer (AJCC), 8th edition (46). Histopathological examination of surgically resected specimens was performed by two pathologists with 10 years of experience in rectal tumor pathology who were blinded to the MRI data. Tumor response to nCRT was classified using the tumor regression grade (TRG) classification system proposed by Mandard et al. (47). Specifically, patients were categorized as pCR if TRG = 1, or non-pCR if TRG > 1.

### Imaging dataset and feature extraction

Patient’s MR images acquired with the 1.5 Tesla MR scanner at Unity MR-Linac during the online adaptive clinical workflow were used for features extraction. The routine scanning protocol consists of 3D-T2-weighted transversal sequences, with image resolution of 0.83 mm × 0.83 mm, slice thickness of 1.5 mm, and acquisition time of 117 s. For each patient, a total of five MR scans, corresponding to fractions 1 to 5, were included in the analysis. Each MRI image was normalized using Z-score normalization method. In addition, to address the issue of high-frequency MRI signal noise and mitigate the impact of significant signal variation, the Laplacian of Gaussian (LoG) filters were applied to raw MRI images. The LoG filters utilized a range of standard deviations (σ) from 0.2 to 1.0 mm, with a step size of 0.2 mm. The calculation is as follows:


$$\text{LoG}\left(\text{x},\text{y}\right)=-\frac{1}{{\text{πσ}}^{4}}\left[1-\frac{x^{2}+y^{2}}{2{\text{σ}}^{2}}\right]e^{-\frac{x^{2}+y^{2}}{2{\text{σ}}^{2}}}$$

**Figure 1:**
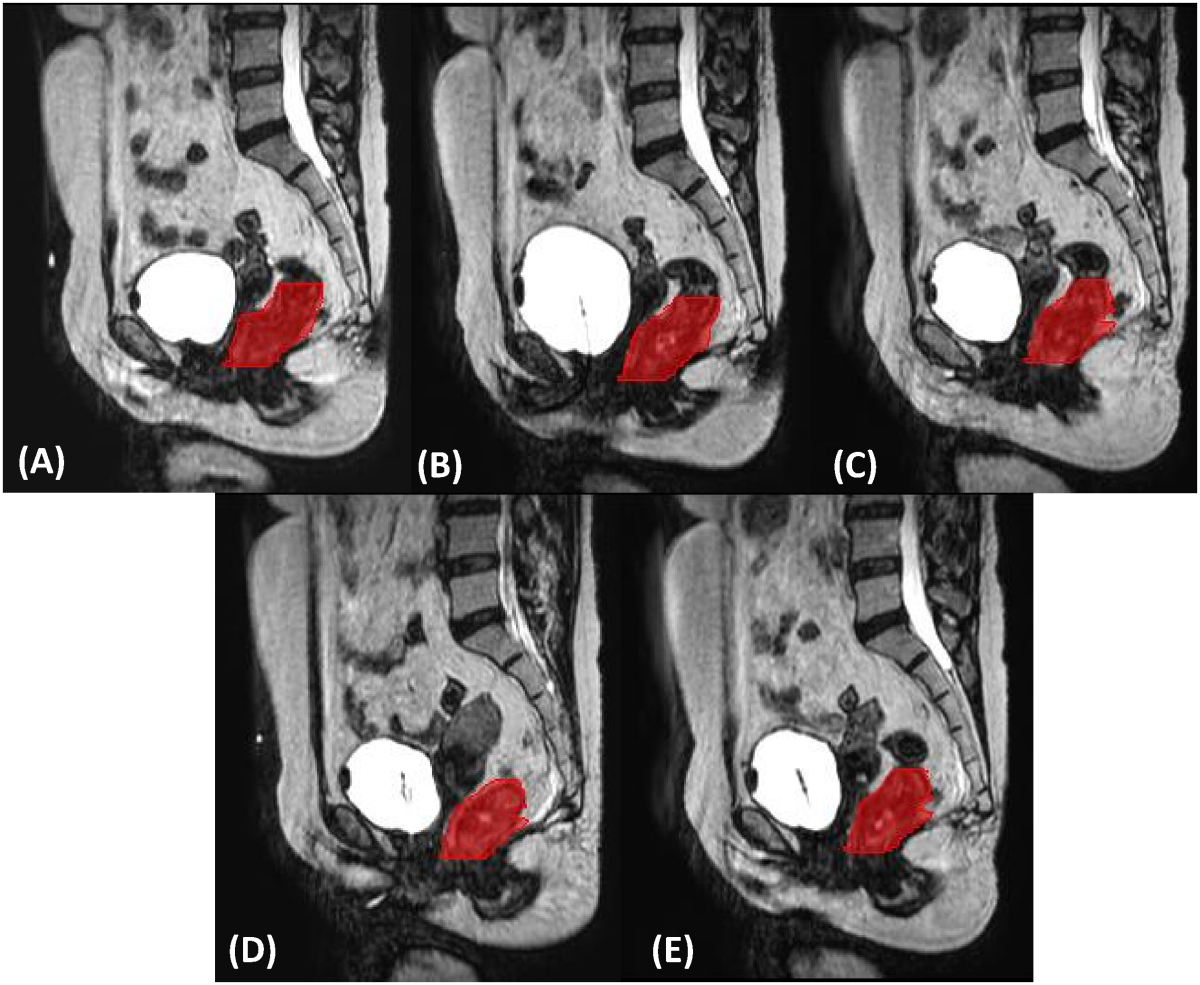
Gross tumor volume delineation (in red) on sagittal planes for the five (**(A–E)**, respectively) MRgART fractions of a representative SCRT.

The segmentation of the gross tumor volume (GTV) used for features extraction is obtained at the daily plan adaptation using first the deformable registration module of Monaco treatment planning system and then a slice-by-slice manual adjustment by the radiation oncologist. The radiomics analysis was focused on the whole GTV volume for each of the five fractions. GTV delineation for a representative patient along the treatment course is shown in **Figure 1**.

Seven families of radiomics features, including First Order, Shape, GLCM, GLDM, GLRLM, GLSZM, and NGTDM, were extracted from both the raw images and the LoG-filtered images. In order to capture changes in the features throughout the treatment, the delta features (ΔF) were calculated as the ratio of the features acquired at fractions 2–5 (F2–F5) to the corresponding features at fraction 1 (F1) as follows, resulting in four datasets (ΔF2, ΔF3, ΔF4, and ΔF5). Additionally, three potential clinical variables, namely clinical T-stage (cT), clinical N-stage (cN), and age, were also collected for subsequent analysis.


$$\text{Δ}F_{i}=F_{i}/F_{1}$$

Where *F*_1_ represents the feature extracted from MRI of the first fraction, *F*_1_ is the features from MRI of *ith* fraction.

### Feature selection and radiomics signature

Firstly, the Mann-Whitney U test was employed to identify potential predictive features (*p*< 0.05) capable of discriminating between patients showing pCR and non-pCR. The test was applied to features, delta features extracted and on the three clinical variables (cN, cT, and age of the patient).

To further reduce the dimensionality of the feature set, the least absolute shrinkage and selection operator (LASSO) was utilized to select the most significant features; the regularization process shrinks the coefficients associated with each variable toward zero, thus retaining only the most relevant features while discarding the less important ones. This was achieved by increasing the regularization factor λ and applying the leave-one-out cross-validation (LOOCV) to determine the optimal set of features. Ultimately, the features and their corresponding coefficients that yielded the lowest “Binomial Deviance” in LASSO were used to construct the radiomics signature (Rad-score) as follows:


$$Rad-score=\sum_{i}^{n}C_{i}X_{i}+\text{b}$$

Where *n* is the number of features, *X* **_i_** and *C* **_i_** are the ith feature and their corresponding coefficients, and b is the intercept.

### Statistical analysis

The MRI images were preprocessed in a Python environment, and features extraction was performed using the PyRadiomics V3.0.1 Python package. Subsequently, the Mann-Whitney U test of the image features, LASSO regression, and evaluation were conducted using R statistical software (version 3.6.1, R Foundation for Statistical Computing, Vienna, Austria).

## Results

### Patients and treatment characteristics

The median age of the 28 patients included in this study was 52.5 years with a higher proportion of males than females (78.6% vs. 21.4%, respectively). Among the patients enrolled, 26 (92.9%) were identified as non-cCR patients and underwent TME surgery at the end of chemotherapy. Of the two cCR patients (7.1%), one remained cCR for 22 weeks after the end of nCRT and then tumor recurrence was detected and underwent surgery; the second one, 45 weeks after the end of nCRT was still cCR at the time of the study, so no pCR information was available. By the end, 27 patients had already undergone surgery and histopathological examination revealed that five of them (18.5%) were pCR. For this radiomics study, 135 MRI datasets and corresponding GTV segmentations of 27 of the 28 LARC patients treated with MRgART at Unity MR-Linac for a SCRT from August 2021 to date were analyzed. The clinical characteristics and response to treatment of the patients are summarized in **Table 1**.

**Table 1:** Patient clinical and treatment characteristics.

Patient number	28
Age – yr.	
Median	52.5
Range	32–71
Sex – no. (%)	
Male	22 (78.6)
Female	6 (21.4)
Tumour stage – no. (%)	
T3	23 (82.1)
T4	5 (17.9)
N0	3 (10.7)
N1	16 (57.1)
N2	9 (32.2)
cCR	
Patient number (%)	2 (7.1)
Interval end CRT- cCR pt1 (weeks)	22
Interval end CRT- cCR pt2 (weeks)	Ongoing still cCR at 45 weeks
Non-cCR	
Patient number (%)	26 (92.9)
Interval-end CRT surgery weeks median range	5.0 (0.1–8.9)
pCR	
Patient number (%)	5 (18.5)
Non-pCR	
Patient number (%)	22(81.5)

### Features selection

A total of 581 features were extracted from each MRI image, including 111 features from the raw image and 470 features from the LoG-filtered images. As for delta features, a total of 2,324 delta features were calculated from the 581 features of each of the four fractions following the first.

The Mann-Whitney U test revealed that 18 variables had a significant association with pCR (*p*< 0.05). These variables included six features, one clinical variable (cN), and 11 delta features (six delta features at second fraction, four delta features at third fraction, one delta feature at fourth fraction). No significant delta features were found at fifth fraction. A standard deviation of 0.2 mm or 0.4 mm of the LoG-filtered images contributed to the extraction of significant features; the results obtained are shown in **Table 2**.

**Table 2:** Features, delta features, and clinical variable significant (*p*< 0.05) to pCR.

Features		σ of LoG (mm)	*p* value				
Name	Class		F1	ΔF2	ΔF3	ΔF4	ΔF5
cN	Clinical	–	0.048	–	–	–	–
Zone entropy	GLSZM	0.2	0.023				
Gray-level variance	GLRLM	0.2	0.033				
Compactness 1	Shape	–	0.047				
Sphericity	Shape	–	0.047				
Spherical disproportion	Shape	–	0.047				
Gray-level non-uniformity normalized	GLRLM	0.2	0.047				
Range	First Order	–		0.008			
Gray-level non-uniformity normalized	GLSZM	–		0.006			
Gray-level variance	GLSZM	–		0.002			
High gray-level zone emphasis	GLSZM	–		0.006			
Small area high gray-level emphasis	GLSZM	–		0.005			
Busyness	NGTDM	–		0.006			
Median	First Order	0.2			0.049		
Cluster shade	GLCM	0.2			0.047		
Gray-level non-uniformity	GLSZM	0.2			0.002		
Size zone non-uniformity	GLSZM	0.2			0.013		
Gray-level non-uniformity	GLSZM	0.4				0.023	

F1, feature at fraction 1; ΔFi, delta feature at fraction i; σ, standard deviation of the LoG-filtered images.

**Figure 2:**
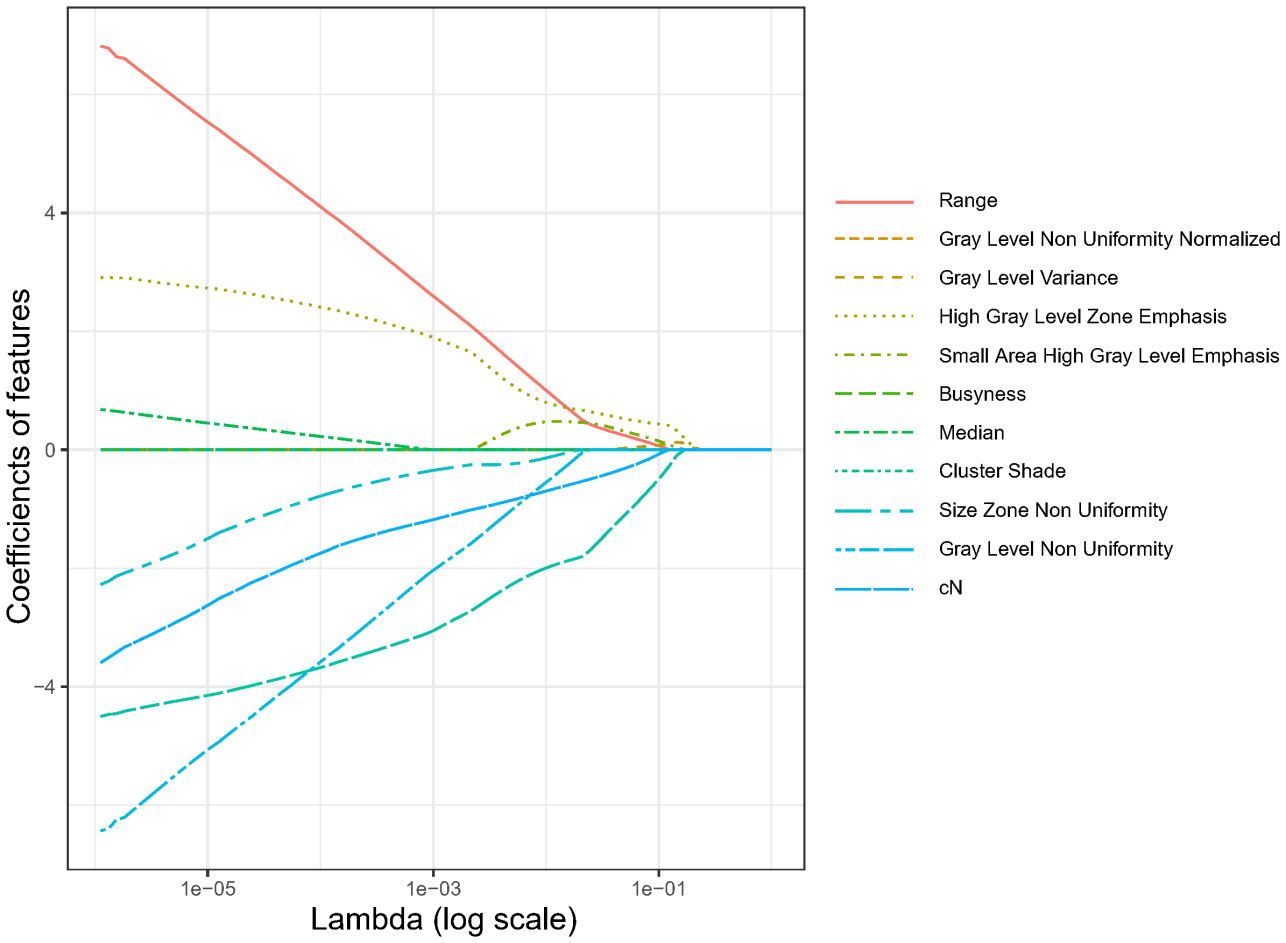
Features dimensionality reduction for increasing values of lambda in LASSO regression.

**Figure 3:**
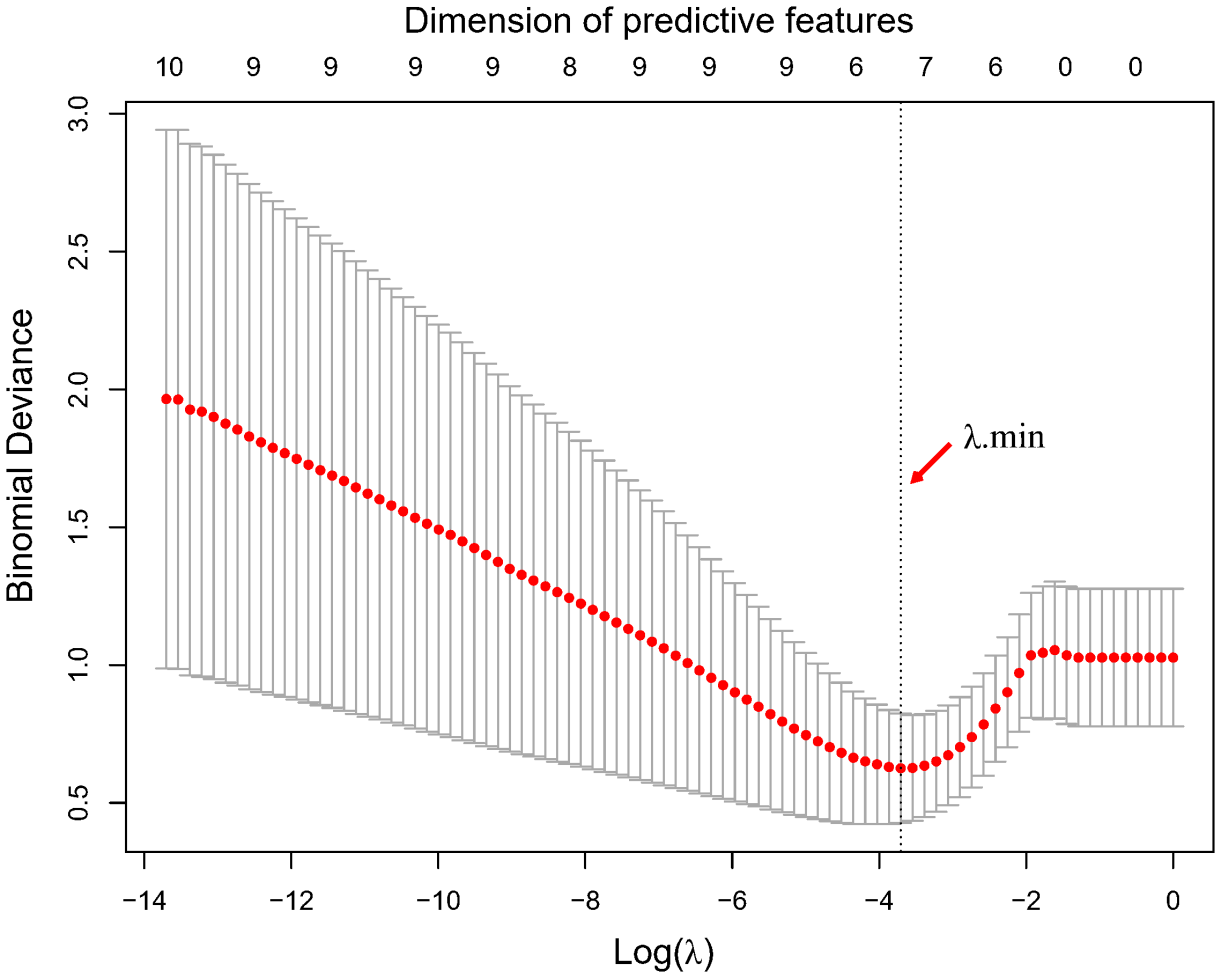
Optimal lambda value determination by minimization of the binomial deviance. The optimal lambda value and corresponding dimension of predictive features with non-zero coefficients are highlighted by the vertical dotted line.

Using LASSO regression, the dimension of the 18 variables was further reduced, thus retaining only the most relevant features and discarding the less important ones. **Figure 2** illustrates the reduction in feature dimensionality for increasing values of Lambda. The binomial deviance of the LASSO regression varies with the regularization parameter lambda. The optimal lambda value (Λ.min) of 0.0245 was obtained when the LASSO regression achieved the lowest binomial deviance using LOOCV validation as shown in **Figure 3**.

**Figure 4:**
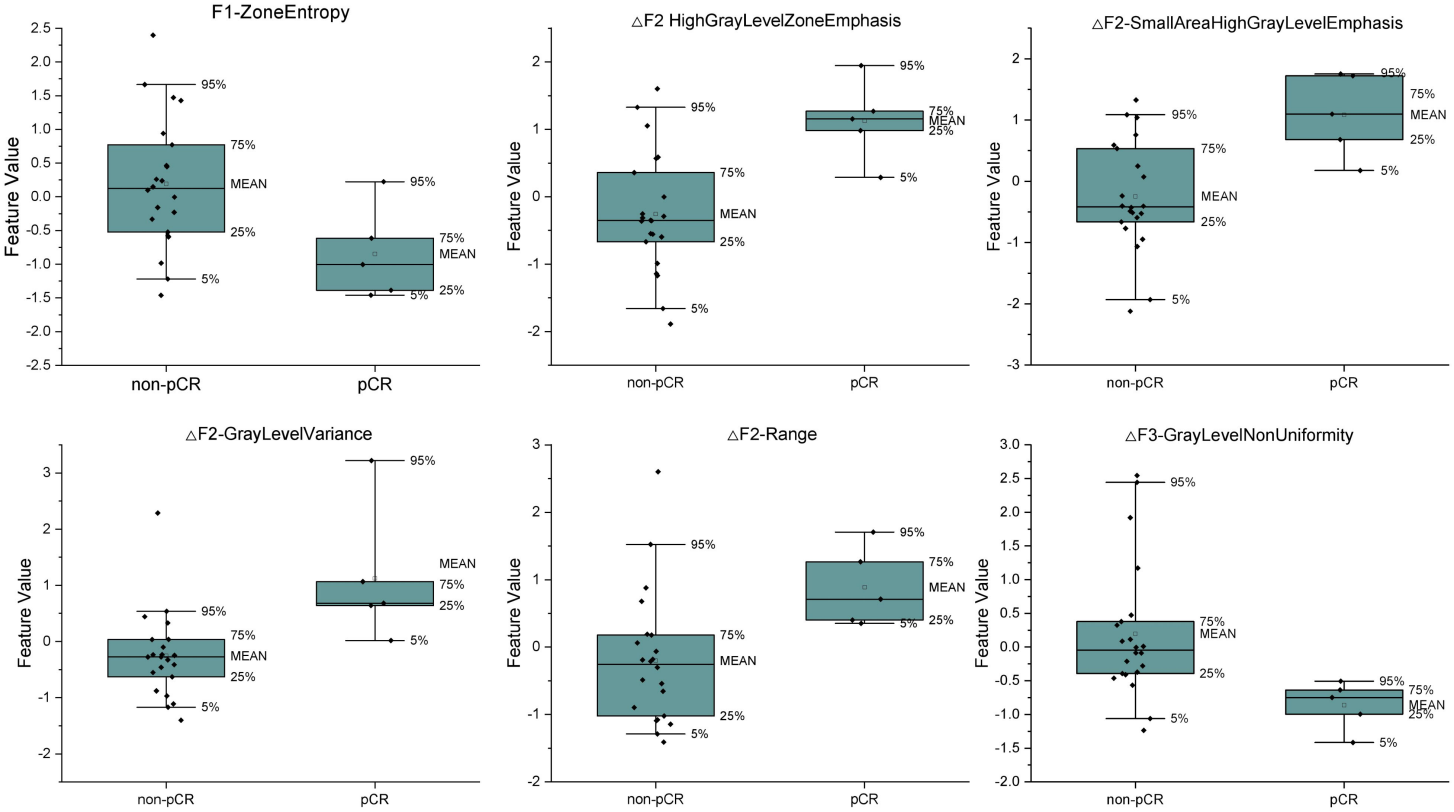
The distributions of the final predictive features and delta features characterizing the pCR and non-pCR patient group. The lower and upper borders of the box indicate the 25th and 75th percentile of the data, respectively, while the whiskers indicate the fifth and 95th percentile of the data.

With this optimal lambda value, 11 features had a zero coefficient in the LASSO regression and were eliminated; of the seven remaining ones, we obtained one clinical variable (cN), one feature from first fraction, four delta features at second fraction, and one delta feature at third fraction, respectively. The final set of significant features and the corresponding coefficients to construct the rad score for patients are listed in **Table 3**. The distributions of all final predictive features characterising the pCR and non-pCR patient groups are shown in **Figure 4**.

**Table 3:** The optimal set of features and corresponding coefficients used to construct the rad score for patients.

Feature			σ of LoG (mm)	Coefficients
Name	Class	Delta category		
cN	Clinical	–	–	−0.184
Zone entropy	GLSZM	F1	0.2	−0.564
Gray-level variance	GLSZM	ΔF2	–	0.058
High gray-level zone emphasis	GLSZM	ΔF2	–	0.462
Small area high gray-level emphasis	GLSZM	ΔF2	–	0.216
Range	First order	ΔF2	0.2	0.139
Gray-level non-uniformity	GLSZM	ΔF3	0.2	−0.748

F1, feature at fraction 1; ΔFi, delta feature at fraction i; σ, standard deviation of the LoG-filtered images.

### Radiomics signature

**Figure 5:**
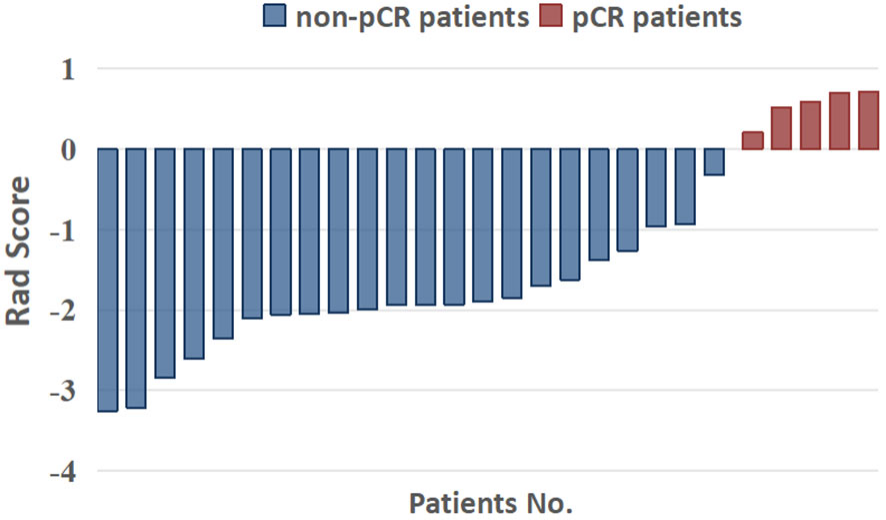
Capability of radiomics signatures (rad score) to significantly distinguish pCR and non-pCR patients.

With the seven variables and their corresponding coefficients, the Rad-Score for each patient was constructed by LASSO regression. **Figure 5** shows that the Rad-Score can significantly discriminate pCR and non-pCR patients (*p*< 0.05).

## Discussion

Artificial Intelligence has gained significant attention in recent years for its potential contribution to achieving full-personalized medicine (48); in the treatment of cancer patients, its role is fundamental throughout the whole process, not only to automatize and speed up the segmentation process, to set up automatic prediction models for the gamma passing rate of the patient’s treatment plan (49, 50), but also in the massive use and analysis of diagnostic imaging to elaborate predictive models for patient’s outcomes. In the management of LARC, personalized medicine is essential; the W&W approach is considered a favorable option for patients assessed as cCR after nCRT to avoid morbidity and toxicity associated with unnecessary surgery. However, the definitive determination of residual tumors relies on the evaluation of resected specimens. Therefore, the ability to predict pCR without the need for an actual total TME procedure would greatly facilitate the personalized treatment approach. Patients predicted to achieve pCR based on reliable models would have a stronger rationale to choose the W&W strategy, whereas those anticipated as non-pCR cases may contemplate additional medical interventions, such as consolidation chemotherapy and/or radiotherapy boost.

Delta radiomics is developed upon conventional radiomics analysis while taking the variation of features throughout the treatment process into consideration. Since radiomics features before or after treatment are not sufficient to describe all characteristics of tumor, the delta radiomics approach might be potentially more powerful for prognostic purposes. MRgART workflow at MR-Linac provides favorable resources for delta radiomics study because multiple MRI images are available for each radiotherapy fraction.

In this study, the delta radiomics approach using the Mann-Whitney U test, Pearson coefficient analysis and LASSO regression was applied to construct the radiomics signature, to identify pCR in a cohort of LARC patients treated in a SCRT at 1.5 Tesla MR. Finally, one original feature and five delta features are selected as the final predictors. Our finding suggests that delta features have the potential to be powerful predictors of pCR for LARC. Other studies also found the potential of delta radiomics when applied to various clinical end points for LARC patients. Boldrini et al. also found that delta features have better discrimination ability than standard radiomics features, even if their study is conducted on the prediction of cCR for LARC. Chiloiro et al. (44) found that the change of area/surface ratio is most significant in identifying 2-year-DFS for LARC. Shayesteh et al. (43) also proved that their delta radiomics model outperformed both pre- and post-treatment features in pCR prediction.

Repeatability and generalization are major concerns in radiomics studies, particularly for delta radiomics studies; heterogeneity in the acquisition of images and timing are important factors to consider (8, 39, 41, 44, 51–54). Those facts might limit the transferability of the results. In our study, delta radiomics analysis was based on MRI from an MR-Linac, allowing reproducibility of the MRI sequences used during the treatment itself and a timing that is guided by the treatment delivery days, and therefore easily reproducible within the same center, and hopefully for multicenter studies (8, 37).

The study is subject to limitations. The number of patients enrolled is too small to set up a fine-tuned prediction model; there is difficulty in performing internal and external validations, reflecting the limited number of LARC patients treated with the relatively new MRgART technique. Moreover, considering that, for each patient, the MRI images were acquired during the SCRT over 1 week, the change in tumor heterogeneity and texture might not be fully exhibited; thus, the change of features might be still conservative. Despite these limitations, the study is the first to investigate and highlight the feasibility of using delta radiomics with promising prediction results on MR-Linac in SCRT schedule for LARC. For a more comprehensive delta radiomics study, future works are under design to include in the analysis one or more MRIs at the end of the SCRT, the continuous updating of the case history, and including internal and external validations as soon as numbers allow.

## Conclusion

Delta features approach based on MRI images acquired during MRgART at MR-Linac could potentially be used for treatment response prediction in LARC patients undergoing nCRT with SCRT.

## Data availability statement

The raw data supporting the conclusions of this article will be made available by the authors, without undue reservation.

## Ethics statement

This research was approved by Ethics Committee of Sichuan Cancer Hospital with the approval number SCCHEC-02-2022-164.

## Author contributions

BT, LO, and QP contributed to conception and design of the study. JX and LO organized the database. YL, FW, and CL performed the statistical analysis. JW and JX wrote the first draft of the manuscript. All authors contributed to the article and approved the submitted version.
